# Characteristics of Atrial Fibrillation Patients Suffering Esophageal Injury Caused by Ablation for Atrial Fibrillation

**DOI:** 10.1038/s41598-020-59539-6

**Published:** 2020-02-17

**Authors:** Pei Zhang, Yue-Yue Zhang, Qian Ye, Ru-Hong Jiang, Qiang Liu, Yang Ye, Jia-Guo Wu, Xia Sheng, Guo-Sheng Fu, Yong-Mei Cha, Chen-Yang Jiang

**Affiliations:** 1 Department of Cardiology, Sir Run Run Shaw Hospital, Zhejiang University School of Medicine, Hangzhou, China; 2 Department of Internal Medicine, Mayo Clinic, Rochester, MN USA; 3 Department of Cardiovascular Medicine, Mayo Clinic, Rochester, MN USA; 4 Department of Gastroenterology, Sir Run Run Shaw Hospital, Zhejiang University School of Medicine, Hangzhou, China

**Keywords:** Gastrointestinal system, Interventional cardiology, Outcomes research

## Abstract

The close proximity of esophagus to the left atrial posterior wall predisposes esophagus to thermal injury during catheter ablation for atrial fibrillation (AF). In this retrospective study, we aimed to investigate risk factors of esophageal injury (EI) caused by catheter ablation for AF. Patients who underwent first-time AF ablation from July 2013 to June 2018 were included. The esophagus was visualized by oral soluble contrast during ablation for all patients and a subset of patients were selected to undergo endoscopic ultrasonography (EUS) to estimate EI post ablation. Degree of EI was categorized as Kansas City classification: type 1: erythema; type 2: ulcers (2a: superficial ulcers; 2b: deep ulcers); type 3: perforation (3a: perforation without communication with the atria; 3b: atrioesophageal fistula [AEF]). Of 3,852 patients, 236 patients (61.5 ± 9.7 years; male, 69%) received EUS (EUS group) and 3616 (63.2 ± 10.9 years; male, 61.1%) without EUS (No-EUS group). In EUS group, EI occurred in 63 patients (type 1 EI in 35 and type 2 EI in 28), and no type 3 EI was observed during follow up. In a multivariable logistic regression analysis, an overlap between the ablation lesion and esophagus was an independent predictor of EI (odds ratio, 21.2; 95% CI: 6.23–72.0; *P* < 0.001). In No-EUS group, esophagopericardial fistula (EPF; n = 3,0.08%) or AEF (n = 2,0.06%) was diagnosed 4–37 days after ablation. In 3 EPF patients, 2 completely recovered with conservative management and 1 died. Two AEF patients died. Ablation at the vicinity of the esophagus predicts risk of EI. EUS post ablation may prevent the progression of EI and should be considered in management of EI. It remains challenging to identify patients with high risk of EI.

## Introduction

Pulmonary vein isolation (PVI) has emerged as a cornerstone therapy for atrial fibrillation (AF) ablation. The close proximity of esophagus to the left atrial posterior wall predisposes esophagus to thermal injury during catheter ablation for AF and Esophageal perforation to the left atrium is a fatal complication^1–5^. Introduction of contact force-sensing catheters and cryo-balloon catheters appears to make no change in procedural complication rates for patients undergoing AF ablation^6^, and the utility of contact force-sensing (CF-sensing) catheter may be associated with increased rates of atrioesophageal fistula (AEF) formation^7^. A recent study appreciably suggested that postprocedural gastroesophageal endoscopy (GSE) could identify EI, and higher intraesophageal temperature measured by a luminal esophageal temperature probe (LET) was associated with the occurrence of EI^5^. However, the protective effect of LET remains controversial since LET itself may also cause EI^8,9^, and routine GSE post AF ablation was not available. Until now, there is no widely accepted approach to minimize esophageal injury (EI) caused by catheter ablation for AF. We sought to identify risk factors of EI caused by ablation for AF, and explore the potential management of EI in this study.

## Methods

### Patients population

All patients with symptomatic paroxysmal or persistent AF undergoing first-time catheter ablation (Radiofrequency or Cryo ablation) at our institution between July 2013 and Jun 2018 were enrolled in a comprehensive database, and the index of procedure was collected and analyzed. The patients were separated into 2 groups: patients with endoscopic ultrasonography (EUS group) and patients without EUS (No-EUS group). All methods were carried out in accordance with relevant guidelines and regulations. The study protocol was approved by the Institutional Review Boards of Sir Run Run Shaw Hospital and written informed consent was obtained from each patient.

### Atrial fibrillation ablation procedure

Transesophageal echocardiography or left atrium (LA) computed tomographic (CT) scan was performed prior to the ablation procedure to rule out left atrial thrombi in all patients. Intravenous heparin 100 IU/kg was administered after successful transseptal puncture, followed by 1,000 IU/hour irrespective of oral anticoagulation regimen. During the procedure, patients received conscious sedation using midazolam and continuous fentanyl infusion according to our standard protocol. LET are not available in our institution.

PVI was a standard approach in all patients. Radiofrequency (RF) ablation was performed using a 3-dimensional electroanatomic mapping system (CARTO; Biosense Webster, CA or NavX/Velocity; Abbott, St. Paul, MN). After double transseptal punctures, the irrigated RF ablation catheter and a circular mapping catheter was positioned into the LA. Radiofrequency energy was delivered at a distance of 5–10 mm from the PV ostia using power control mode (35 W for the anterior wall, and down to 25 W for the posterior wall) as previous described^10^. When there was an overlap between the ablation lesion and esophagus along the posterior wall, modified ablation was applied (power ≤25 W and duration ≤15 seconds)^11^. Additional ablation procedures, such as Box lesion for persistent AF, were performed at the discretion of the operators.

Cryo ablation was performed using a 23/28-mm cryoballoon (Arctic Front Advance, Medtronic, Inc., Minneapolis, MN) inserted via a 12 French steerable sheath (FlexCath, Medtronic, Inc.) over a circular inner lumen mapping catheter/guidewire (Achieve, Medtronic, Inc.). One to two 120- to 180-second cryo applications were delivered to each PV guided by time to PVI. Modified ablation was applied (temperature ≥−50 °C and shorter duration [≤120 s when time to PVI less than 60 s]) when there was an overlap between the ablation lesion and esophagus^12^. PVI was assessed continuously using the circular Achieve catheter during cryoablation. If PV remained connected after 2 or more cryo applications, RF application was performed to achieve PVI using wide antral circumferential ablation technique.

### Overlap between the ablation lesion and esophagus course

**Figure 1:**
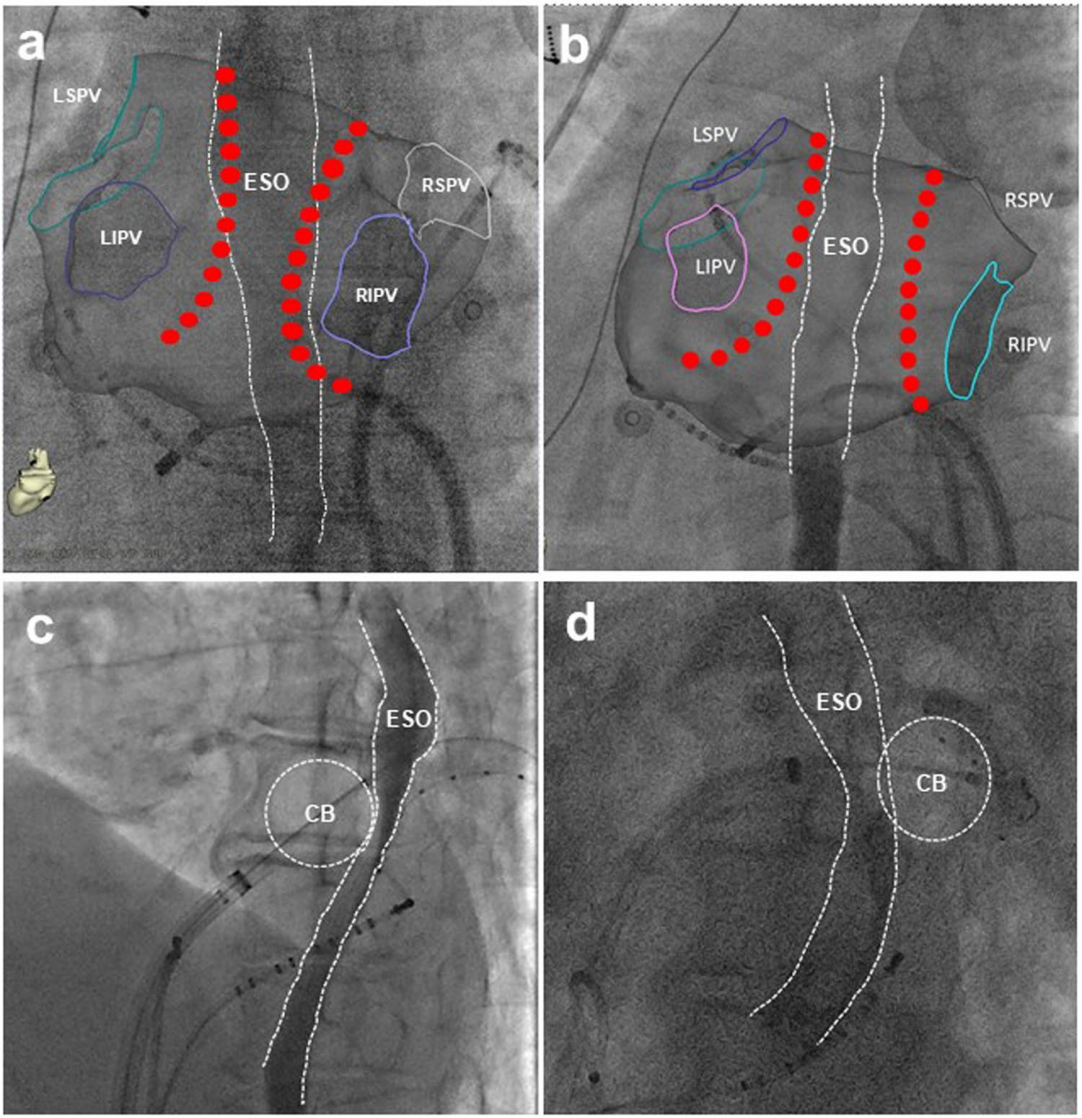
Overlap between the ablation lesion and esophagus course. The designed radiofrequency ablation lesion with (**a**, PA view) and without (**b**, PA view) overlap with esophagus course. Cryo ablation lesion with (**c**, LAO view) and without (**d**, LAO vies) overlap with esophagus course. CB = cryoballoon; ESO = esophagus.

Before ablation energy was delivered, every patient was given oral soluble contrast (5 ml) to determine the esophagus course under fluoroscopy, and the ablation lesion was designed to minimize direct contact of ablation catheter with the esophagus^10^. The spatial relationship between the esophagus and left atrial posterior wall was evaluated in posterior-anterior (PA) and left lateral (LL) projection views during RF ablation and two orthogonal projection views of left anterior oblique (LAO) and right anterior oblique (RAO) during cryo ablation. When the ablation lesion points was beyond the esophagus margin (Fig. 1A) or the anterior half of cryoballoon was near the esophagus under LAO (Fig. 1C), the overlap between ablation lesions and esophagus course was determined (Fig. 1).

### Esophageal endoscopy and ultrasonography

The decision to perform EUS was made at the discretion of the operators based on potential risk factors of EI^3,13–17^, as well as clinical manifestation post ablation (such as fever, chest discomfort, elevated white blood cell count and C-reaction protein level).

EUS was performed by experienced operators to assess the magnitude of EI. EI was defined as any esophageal lesion adjacent to the contact area between the esophagus and the LA, and was defined according to Kansas City classification: type 1: erythema; type 2: ulcers (2a: superficial ulcers; 2b: deep ulcers); type 3: perforation (3a: perforation without communication with the atria; 3b: perforation with atrioesophageal fistula)^18^. All gastroscopies (GIFQ 260, GIFQ 165, GIFQ 145; Olympus, Japan) were performed by endoscopists in an endoscopy laboratory. A radial echoendoscope (EU-ME2 PREMIER PLUS, Olympus, Japan) was used for examination. Careful examination of the mediastinum and esophageal wall was performed to assess mucosal and periesophageal/mediastinal lesions. Patients were kept fasting for at least 8 hours prior to the procedure. Examinations were performed in the left lateral decubitus position and under conscious sedation with propofol.

### Management of esophageal injury

**Figure 2:**
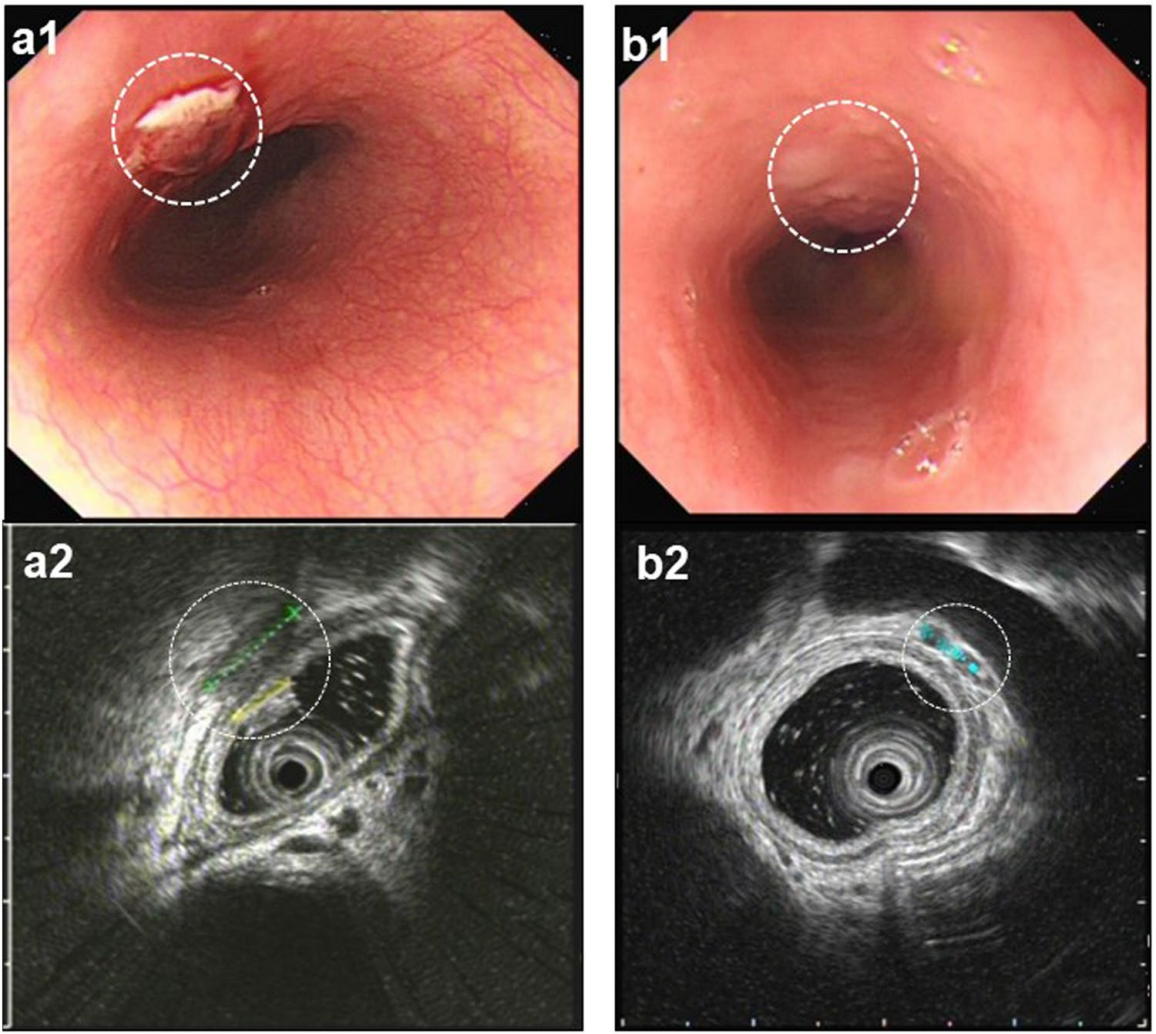
Esophageal injury post ablation shown by endoscopic ultrasonography. Gastroscopy revealed esophageal ulcer (**a1**, white dot circle) 2 days after radiofrequency ablation. Ultrasonography displayed the thickening and loss of the submucous layers architecture (**a2**, white dot circle). The esophagus recovered with normal architecture 10 days later (**b1**, **b2**, white dot circle).

All patients received routine soft diet and proton pump inhibitors (PPI) for 6 weeks after AF ablation. Patients with type 1 EI received semi solid diet as well as PPI. Patients with type 2 EI were given intravenous PPI and kept fasting until repeat EUS showed resolution of EI or improvement to type 1 lesion (Fig. 2). If expected recovery time was beyond 7 days, a jejunal feeding tube was placed.

Once type 3 EI was suspected, contrast enhanced CT scan was performed. Type 3 EI was determined when imaging showed extravasation of air or oral contrast medium from the esophagus to the mediastinum, pericardium or LA. When AEF was ruled out, EUS was required to evaluate the magnitude of EI. For patients with esophagopericardial fistula (EPF), timely pericardiocentesis and drainage were performed in addition to adjunctive treatment with broad spectrum antibiotics and nutritional support. Once AEF was diagnosed, multidisciplinary evaluation took place and surgical repair was considered.

### Follow up

All patients were followed every 2 weeks in the first 3 months after AF ablation and every 3 months thereafter.

### Statistical analysis

Continuous variables are expressed as mean ± SD or median. ANOVA was used to compare different groups. Categorical variables are presented as counts and percentages and were compared across groups using the χ2 test or Fisher exact test, as appropriate. For logistic regression, results were given as odds ratios, 95% confidence intervals and *P* values. *P* values < 0.05 was considered statistically significant. Statistical analyses were performed using SPSS version 23 (IBM).

## Results

### Patients population

**Figure 3:**
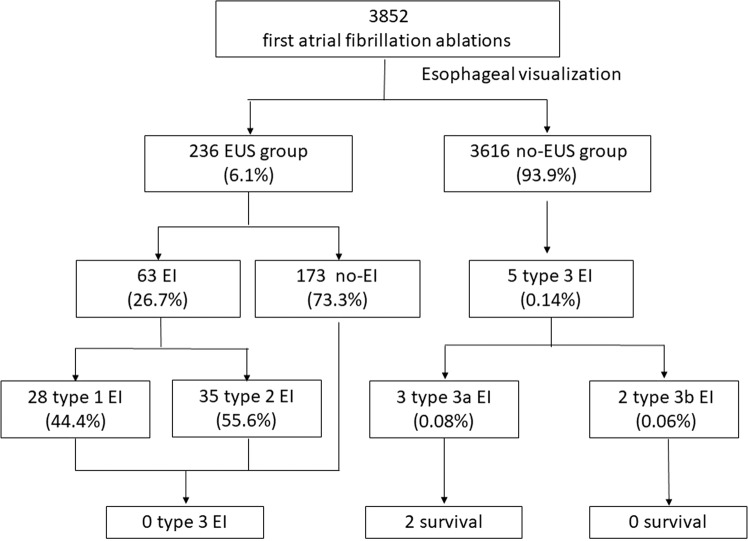
Flow chart of patients cohort analyzed and included in the study. EI = esophageal injury; EUS = endoscopic ultrasonography; Kansas City classification: type 1: erythema; type 2: ulcers (2a: superficial ulcers; 2b: deep ulcers); type 3: perforation (3a: perforation without communication with the atria; 3b: AEF).

A total of 3,852 patients underwent PVI for first-time AF ablation at our institution between July 2013 and June 2018. Of these, 236 patients (61.5 ± 9.7 years; male, 69.1%) underwent EUS within 3 days after ablation (EUS group) and 3,616 patients (63.2 ± 10.9years; male, 61.1%) did not undergo EUS (No-EUS group, Fig. 3). The patients in EUS group had lower BMI and higher percentage of hypertension and diabetes compared to those in No-EUS group (Table 1).

**Table 1:** Baseline characteristic of patients undergoing ablation of AF.

		EUS group (n = 236)	No-EUS group (n = 3616)	P value
Age (y)		61.5 ± 9.7	63.2 ± 10.9	0.02
BMI		24.2 ± 2.8	25.1 ± 3.0	<0.001
CHA2DS2-VASC Score		1.5 ± 1.5	1.6 ± 1.4	0.27
Male, n (%)		163 (69.1)	2212 (61.2)	0.014
Paroxysmal AF, n (%)		172 (72.9)	1991 (55.1)	<0.001
Hypertension, n (%)		122 (51.7)	1024 (28.3)	<0.001
Diabetes, n (%)		25 (10.6)	221 (6.1)	0.006
Stroke, n (%)		10 (4.2)	98 (2.7)	0.16
CAD, n (%)		16 (6.8)	216 (6.0)	0.601
Smoke		32 (13.6)	500 (13.8)	0.92
Alcohol		47 (19.9)	797 (22.0)	0.45
LA (mm)		36.6 ± 5.2	36.7 ± 5.1	0.78
LVEF		69.3 ± 7.0	69.5 ± 7.1	0.71
Energy Source	RF	161 (68.2)	3228 (89.3)	<0.001
	Cryo	53 (22.5)	330 (9.1)	
	RF&cryo	22 (9.3)	58 (1.6)	
overlap		150 (63.6)	2046 (56.6)	0.04

AF: atrial fibrillation, BMI: body mass index, CAD: coronary artery disease, EI: esophageal injury, LA: left atrium, LVEF: left ventricular ejection function, Overlap: Overlap between ablation lesion and esophagus course, RF: radiofrequency.

### Esophageal injury in EUS group

In EUS group, 236 patients underwent AF ablation with 3 energy modalities: 161 patients (68.2%) with RF, 53 patients (22.5%) with Cryo and 22 patients (9.3%) with Cryo plus RF. Out of 236 patients, 150 (63.6%) had overlap between ablation lesions and esophagus course and 60 patients (40%) had EI: 25 patients with type 1 EI and 35 with type 2 EI, whereas in 86 patients without overlap between ablation lesion an esophagus, 3 patients (3.5%) had category 1 EI (*P* < 0.001). There was a significant difference in EI occurrence among 3 different ablation energy modalities (*P* = 0.03). In a multivariate logistic regression analysis, the ablation energy modality and overlap between the ablation lesion and esophagus were identified as independent predictors of EI. Compared to RF ablation, cryo ablation produced lower incidence of EI (odds ratio, 0.28; 95% CI: 0.11–0.72; *P* = 0.009; Table 2), and the overlap between ablation lesion and esophagus course predicted higher incidence of EI (odds ratio, 21.2; 95% CI: 6.23–72.0; P < 0.001; Table 2). All the patients with type 2 EI were given intravenous PPI twice daily and kept fasting until repeat EUS showed resolution of EI or improvement to type 1 lesion, and a jejunal feeding tube was placed in 3 patients (8.6%). No category 3 EI was observed during 31 ± 18 months follow-up.

**Table 2:** Characteristic of patients undergoing endoscopic ultrasonography.

		EI group (n = 63)	No-EI group (n = 173)	P Value	Multivariable logistic regression	
					Odds Ratio (95% CI)	P Value
Age, years		61.8 ± 10.0	61.4 ± 9.7	0.81	—	—
Male, n (%)		45 (71.4)	118 (68.2)	0.64	—	—
BMI		23.8 ± 2.8	24.4 ± 2.8	0.12	0.891 (0.789–1.005)	0.06
Paroxysmal AF, n (%)		45 (71.4)	127 (73.4)	0.76	—	—
Hypertension, n (%)		32 (50.8)	90 (52)	0.87	—	—
Diabetes, n (%)		8 (12.7)	17 (9.8)	0.53	—	—
CAD, n (%)		6 (9.5)	10 (5.8)	0.47	—	—
Stroke, n (%)		5 (7.9)	5 (2.9)	0.18	1.786 (0.427–7.427)	0.43
CHA_2_DS_2_-VASc Score		1.6 ± 1.3	1.4 ± 1.2	0.36	—	—
Smoke, n (%)		10 (15.9)	22 (12.7)	0.53	—	—
Alcohol, n (%)		12 (19)	35 (20.2)	0.84	—	—
LA Diameter (mm)		36.3 ± 4.9	36.7 ± 5.3	0.65	—	—
LVEF (%)		70.3 ± 8.0	68.9 ± 6.5	0.17	1.018 (0.97–1.068)	0.48
Energy Source	RF, n (%)	51 (81)	110 (63.6)	0.03	—	0.03
	Cryo, n (%)	7 (11.1)	46 (26.6)		0.279 (0.108–0.723)	0.01
	RF&Cryo, n (%)	5 (7.9)	17 (9.8)		0.615 (0.192–1.97)	0.41
LAPW ablation, n (%)		7 (11.1)	12 (6.9)	0.30	0.972 (0.32–2.95)	0.96
Overlap, n (%)		60 (95.2)	90 (52)	<0.001	21.18 (6.232–71.98)	<0.001
Fever, n (%)		10 (15.9)	21 (12.1)	0.45	—	—
Chest discomfort, n (%)		5 (7.9)	11 (6.4)	0.89	—	—
WBC		8.8 ± 2.4	8.6 ± 3.0	0.56	—	—
CRP (median)		10.5	13	0.79	—	—

AF: atrial fibrillation; BMI: body mass index; CAD: coronary artery disease; CRP: C-reaction protein; EI: esophageal injury; LA: left atrium; LAPW: left atrium posterior wall; LVEF: left ventricular ejection function; Overlap: Overlap between ablation lesion and esophagus course; RF: radiofrequency; WBC: white blood cell.

### Esophageal injury in No-EUS group

**Figure 4:**
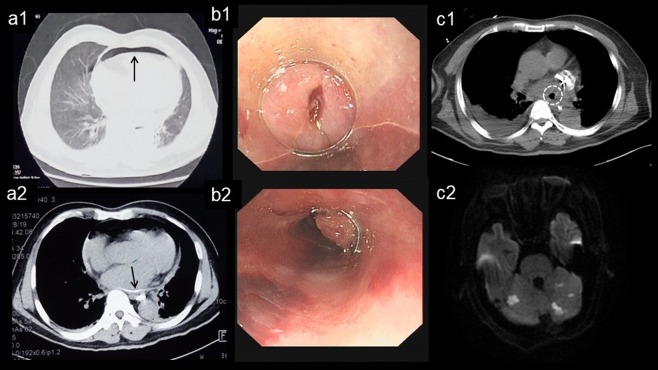
Progression from esophagopericardial fistula to atrioesophageal fistula. Unenhanced CT revealed pneumopericardium (**a1**, arrow) and the presence of Barium sulfate suspension prior esophagraphy within the pericardial sac (**a2**, arrow). Endoscopic closure of esophagus perforation with titanium clips (**b1**,**b2**). The gas (white dot circle) in left atrium documented by CT (**c1**) and multiple cerebral embolism documented by MRI (**c2**).

**Figure 5:**
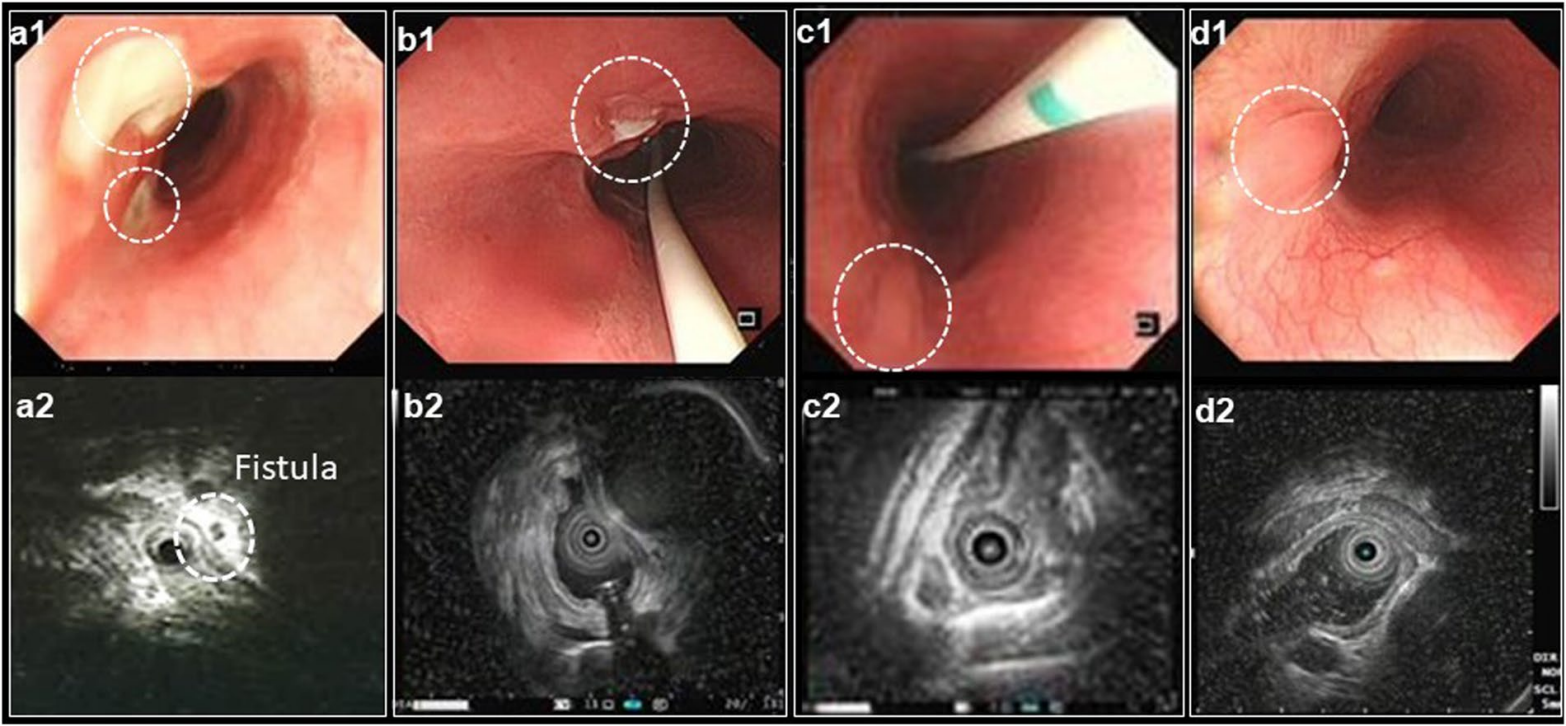
Evolution of the esophagopericardial fistula post ablation. The gastroscopy demonstrated the progression of esophagopericardial fistula 7 days (**a1**) 14 days (**b1**), 21 days (**c1**) and 30 days (**d1**) after RF ablation. Ultrasonography revealed dense echo with air, which indicated local fistula formation (**a2**, white dot circle) and recovery of esophageal tissue layer (**b2**,**c2**,**d2**).

In No-EUS group, 3,616 patients underwent AF ablation with 3 energy modalities: 3227 patients (89.2%) with RF, 331 patients (9.2%) with Cryo and 58 patients (1.6%) with RF plus Cryo. In totally, 2045 patients (56.6%) had overlap between ablation lesions and esophagus course. Five patients (0.14%) developed category 3 EI: 3 with category 3a (EPF) and 2 with category 3b (AEF, Table 3). All 5 patients received RF ablation and had an overlap between ablation lesion and esophagus course. In the 3 patients with EPF, 1 died resulting from progression to AEF after undergoing endoscopic closure with titanium clip (Fig. 4), and 2 recovered without sequelae after conservative management (Fig. 5). In patients with AEF, 1 underwent surgical repair but died due to septic shock, and 1 died 3 days after admission to a local hospital.

**Table 3:** Characteristic of patients with type 3 esophageal injury.

Patient No.	Gender	Age (y)	Type of AF	CHA_2_DS_2_- VASc Score	LVEF (%)	Left Atrium Diameter (mm)	Overlap	Clinical Manifestation	Days after Ablation	Anesthesia	Ablation Catheters	Type of Fistula	Treatment	Fatal
1	M	67	PeAF	3	65	45	Yes	Fever	7	CS	Non-CF	AEF	Conservative	Yes
2	M	72	PeAF	4	59	48	Yes	Fever, Chest Pain	16	GA	Non-CF	EPF	Titanium Clip	Yes
3	M	77	PAF	3	71	38	Yes	Fever, Seizure	4	CS	Non-CF	AEF	Surgery	Yes
4	M	66	PAF	2	65	32	Yes	Fever	37	CS	Non-CF	EPF	Conservative	No
5	F	55	PAF	1	72	36	Yes	Chest Distress	7	CS	Non-CF	EPF	Conservative	No

AEF: atrioesophageal fistula; CS: conscious sedation; EPF: esophagopericardial fistula; F: female; GA: general anesthesia; M: male; Non-CF: non-contact force-sensing; Overlap: Overlap between ablation lesion and esophagus course; PAF: paroxysmal atrial fibrillation; PeAF: Persistent atrial fibrillation.

## Discussion

The study was a retrospective observational study, and the main findings are: (1) Overlap between the ablation lesion and the esophageal course is an independent predictor of EI; (2) EUS may prevent the progression of EI, and conservative treatment guided by EUS may be an alternative in the management of EPF; (3) It remains challenging to identify patients with high risk of EI.

### Incidence and predictors of esophageal injury

A recent meta-analysis demonstrated that the prevalence was 11% and 5% in any and ulcerated EI caused by ablation of AF respectively^9^. In our study, the incidence of EI was 26.7% in EUS group, in which the patients were at high risk of EI, however, this incidence was obviously overestimated due to selection bias. Our previous study showed that the incidence of EI was 5.1% and 20.5% in consecutive patients with esophageal visualization and in those without^10^. A prior study did not demonstrate prevention of EI using esophageal visualization because of low incidence of EI^3^, whereas overlap between ablation lesion and esophagus course was an independent predictor of EI in our study, and EI seldom occurred when the overlap was absent, so we developed a feasible approach to effectively decrease the risk of EI by using soluble oral contrast to visualize the esophagus during the ablation procedure and thereby allowing us to design the ablation lesion away from the esophagus. Consistent with previous study^3^, the strategy of reducing the power at posterior wall did not prolong the time to PVI. The PV reconnections or dormant conduction in areas with direct contact with the esophagus was not observed in our study. Our previous study suggested that modified ablation guided by esophageal visualization could decrease the occurrence of EI and there was no significant difference in AF recurrence during 12-month follow-up^10^.

The latest study indicated that cryoballoon ablation for AF guided by time to PVI reduced the esophageal complications^19^. Consistent with the previous survey on cryoballoon ablation, in which a total of 11 cases of AEF were reported from more than 120,000 cases worldwide^20^, our data supported that cryoballoon ablation had lower incidence of EI, although this is not our main aim. Many signs post ablation, such as fever, chest discomfort, elevated WBC count and CRP level, may be caused by vagal nerve injury or inflammatory reaction. These factors were non-specific and could not be used to identify patients who were at high risk of EI.

The estimated incidence of AEF, at 0.05% to 0.25%^21^, may be underestimated because of under-reporting or misdiagnosis. Halbfass’s study demonstrated that only ulcerative EI was associated with esophageal perforation^5^. Even though the patients with high risk of EI were selected to undergo EUS, 5 patients developed type 3 EI in no-EUS group, suggesting it warrants further investigation to identify reliable predictors of type 3 EI.

### Management of esophagus injury

Although the precise mechanism of EI from ablation is not completely understood, Esophageal ulcer seems to precede AEF development^21,22^. Halbfass’s study indicated about 10% of ulcerative EI progressed to esophageal perforation^5^. Given that the ablation-induced injury affects the esophagus from “outside in” and mucosal visualization can underrepresent the actual magnitude of EI^18^, EUS was performed in patients at high risk of developing EI in our study. In EUS group, no patients with ulcerative EI progressed to type 3 EI resulting from aggressive treatment guided by EUS.

It is noted that the incidence of EPF was higher than that of AEF in our study. Previous survey revealed low incidence of EPF^4^, which may be due to delayed diagnosis of EPF preceding AEF. The first EPF patient developed to AEF after endoscopic fistula closure with titanic clip, suggesting that the PEF may be a transition phase from EI to AEF. Esophageal stent was considered to be an effective treatment for EPF, however, some complications could be caused by the insertion of stent, such as pneumopericardium, stent dislocation and hematemesis^23^. EUS may be useful in detecting submucosal esophageal and mediastinal changes after PVI^24^, which can be used to assess the magnitude of EI and inflammation reaction. Moreover, EUS can be used to evaluate the recovery of all esophageal tissue layers, and therefore to determine the termination of fasting, which is essential and effective in restoring the esophageal continuity and function. The following 2 patients with EPF recovered without sequelae after conservative management guided by EUS. Conservative treatment, including pericardiocentesis and antibiotic therapy, may be an alternative in the management of EPF. Most signs and symptoms of esophageal perforation are not specific. EI without fistula formation to left atrium is associated with better survival outcome^25^, which underlines the importance of diagnosing EI at its earlier stage. In conclusion, we believe EUS should be performed if there is clinical suspicion of earlier EI.

## Limitations

This study had several limitations. First, it was a single-center retrospective observational study and the accurate reasons for performing EUS were not available. Given that risk factors of EI remain controversial, the decision to pursue EUS post ablation was made at the discretion of the operators, and this was a source of selection bias leading to overestimation of the incidence of EI and attenuation of the statistic power on risk factors of EI. Moreover, study results obtained from a patient cohort managed at our institution using our standard protocol may not be applicable to other institutions. Ablation was performed under conscious sedation routinely, and therefore the effect of general anesthesia (GA) was not assessed. In our cohort, category 2 EI (n = 2) and type 3a EI (n = 1) occurred in ablations under GA. In a small randomized study, EI was observed in 48% of patients in GA group and only 4% in the conscious sedation group^26^. GA likely increases the risk of EI probably as a result of enhanced contact and reduced esophageal motility^21^. Contrast based localization of the esophagus is definitely useful but not perfect because esophagus can move during the procedure. Good *et al*. assessed esophageal movement that occurred during catheter ablation for AF under conscious sedation, and results showed that 67% of patients had a shift of ≥2 cm and 4% had a shift of ≥4 cm of lateral movement^27^. We did not evaluate the effect of CF–sensing catheter on EI because CF-sensing catheter was not available until 2017 at our institution and only seldom type 1 EI was observed with the use of these catheters. Finally, because of small number of patients with type 3 EI, we did not assess the statistical difference between patients with and without type 3 EI in No-EUS group.

## Conclusions

Ablation at the vicinity of esophagus increases the risk of EI. EUS post ablation may prevent the progression of EI, and may be useful in the conservative management of PEF. It remains challenging to identify reliable predictors of EI.
